# Decentralized care for multidrug-resistant tuberculosis: a systematic review and meta-analysis

**DOI:** 10.2471/BLT.17.193375

**Published:** 2017-08-01

**Authors:** Jennifer Ho, Anthony L Byrne, Nguyen N Linh, Ernesto Jaramillo, Greg J Fox

**Affiliations:** a Woolcock Institute of Medical Research, University of Sydney, 431 Glebe Point Road, Glebe, New South Wales 2037, Australia.; b Global TB Programme, World Health Organization, Geneva, Switzerland.; c Central Clinical School, University of Sydney, Sydney, Australia.

## Abstract

**Objective:**

To assess the effectiveness of decentralized treatment and care for patients with multidrug-resistant (MDR) tuberculosis, in comparison with centralized approaches.

**Methods:**

We searched ClinicalTrials.gov, the Cochrane library, Embase®, Google Scholar, LILACS, PubMed®, Web of Science and the World Health Organization’s portal of clinical trials for studies reporting treatment outcomes for decentralized and centralized care of MDR tuberculosis. The primary outcome was treatment success. When possible, we also evaluated, death, loss to follow-up, treatment adherence and health-system costs. To obtain pooled relative risk (RR) estimates, we performed random-effects meta-analyses.

**Findings:**

Eight studies met the eligibility criteria for review inclusion. Six cohort studies, with 4026 participants in total, reported on treatment outcomes. The pooled RR estimate for decentralized versus centralized care for treatment success was 1.13 (95% CI: 1.01–1.27). The corresponding estimate for loss to follow-up was RR: 0.66 (95% CI: 0.38–1.13), for death RR: 1.01 (95% CI: 0.67–1.52) and for treatment failure was RR: 1.07 (95% CI: 0.48–2.40). Two of three studies evaluating health-care costs reported lower costs for the decentralized models of care than for the centralized models.

**Conclusion:**

Treatment success was more likely among patients with MDR tuberculosis treated using a decentralized approach. Further studies are required to explore the effectiveness of decentralized MDR tuberculosis care in a range of different settings.

## Résumé

**Objectif:**

Évaluer l'efficacité de la prise en charge et des traitements décentralisés pour les patients atteints de tuberculose multirésistante (TB-MR), par rapport aux approches centralisées.

**Méthodes:**

Nous avons recherché, dans les bases de données de ClinicalTrials.gov, de la Cochrane Library, d'Embase®, de Google Scholar, de LILACS, de PubMed® et de Web of Science, ainsi que sur le portail de l'Organisation mondiale de la Santé dédié aux essais cliniques, des études qui mentionnaient les résultats d’une prise en charge centralisée ou décentralisée de la tuberculose multirésistante. Le premier critère pris en compte était la réussite du traitement. Lorsque cela était possible, nous avons aussi évalué le nombre de décès, l'interruption du suivi, le respect du traitement et les coûts pour le système de santé. Afin d'obtenir des estimations globales du risque relatif (RR), nous avons procédé à des méta-analyses à effets aléatoires.

**Résultats:**

Huit études remplissaient les critères d'admissibilité permettant d'être incluses dans notre revue. Six études de cohortes, comportant 4026 participants au total, mentionnaient les résultats de traitements. L'estimation globale du RR d'une prise en charge décentralisée par rapport à une prise en charge centralisée concernant la réussite du traitement était de 1,13 (IC 95%: 1,01-1,27). Les estimations correspondantes du RR étaient, pour l'interruption du suivi, de 0,66 (IC 95%: 0,38–1,13), pour le décès: 1,01 (IC 95%: 0,67–1,52) et pour l'échec du traitement: 1,07 (IC 95%: 0,48-2,40). Deux études sur trois qui évaluaient les coûts pour le système de santé ont fait état de coûts plus faibles pour les modèles décentralisés que pour les modèles centralisés.

**Conclusion:**

Les traitements avaient plus de chances de réussir chez les patients atteints de tuberculose multirésistante traités selon une approche décentralisée. D'autres études sont nécessaires pour évaluer l'efficacité de la prise en charge décentralisée de la tuberculose multirésistante dans une diversité de contextes.****

## Resumen

**Objetivo:**

Evaluar la efectividad de la atención y el tratamiento descentralizados para pacientes con tuberculosis multirresistente (TB-MR), en comparación con los enfoques centralizados.

**Métodos:**

Se realizaron búsquedas de estudios que informen sobre resultados de tratamientos para la TB-MR con atención descentralizada y centralizada en ClinicalTrials.gov, la Biblioteca Cochrane, Embase®, Google Scholar, LILACS, PubMed®, Web of Science y el portal de ensayos clínicos de la Organización Mundial de la Salud. El resultado principal era el éxito del tratamiento. Cuando fue posible, también se tuvieron en consideración las defunciones, las pérdidas durante el seguimiento, el cumplimiento del tratamiento y los costes del sistema sanitario. Para obtener estimaciones de riesgo relativo (RR) combinadas, se realizaron metaanálisis de efectos aleatorios.

**Resultados:**

Ocho estudios cumplieron con los criterios de elegibilidad para incluirse en la revisión. Seis estudios de cohortes, con 4 026 participantes en total, contribuyeron con los resultados del tratamiento. La estimación de RR combinada de la atención descentralizada frente a la centralizada para el éxito del tratamiento fue de 1,13 (IC del 95%: 1,01–1,27). Las estimaciones correspondientes para las pérdidas durante el seguimiento tuvieron un RR de 0,66 (IC del 95%: 0,38–1,13), el RR de las defunciones fue de 1,01 (IC del 95%: 0,67–1,52) y el RR del incumplimiento del tratamiento fue de 1,07 (IC del 95%: 0,48–2,40). Dos de tres estudios que evaluaban los costes de la atención sanitaria registraron menos costes para los modelos de atención descentralizada que para los modelos centralizados.

**Conclusión:**

El éxito del tratamiento era más probable entre pacientes con TB-MR tratada con un enfoque descentralizado. Los estudios adicionales necesitan explorar la efectividad de la atención descentralizada de la TB-MR en una variedad de distintos contextos.

## ملخص

**الغرض:**

تقييم فعالية العلاج اللامركزي ورعاية المرضى الذين يعانون من مرض السل المقاوم للأدوية المتعددة (MDR)، بالمقارنة مع الأساليب المركزية.

**الطريقة:**

قمنا بالبحث في ClinicalTrials.gov، ومكتبة Cochrane، وEmbase®‎، وGoogle Scholar، وLILACS، وPubMed®‎، وWeb of Science والبوابة الإلكترونية لمنظمة الصحة العالمية الخاصة بالتجارب السريرية عن دراسات إبلاغ نتائج علاج الرعاية اللامركزية والمركزية لمرض السل المقاوم للأدوية المتعددة. وأظهرت النتيجة الأولية نجاح العلاج. كما قمنا بتقييم الوفيات، وفقدان المتابعة، والالتزام بالعلاج، وتكاليف النظام الصحي عندما كان ذلك ممكنًا. وللحصول على تقديرات الاختطار النسبي المجمعة (RR)، أجرينا تحاليل تلوية عشوائية التأثيرات.

**النتائج:**

استوفت ثماني دراسات معايير الأهلية لإدراجها في عملية المراجعة. وقد أوردت ست دراسات أترابية عن نتائج العلاج والتي ضمت 4026 مشاركًا بشكل إجمالي. وكانت نسبة نجاح علاج التقدير المجمع للاختطار النسبي من أجل الرعاية اللامركزية مقابل الرعاية المركزية 1.13 (بنسبة أرجحية مقدارها 95‏%: 1.01–1.27). وكانت التقديرات المقابلة لفقدان المتابعة هي الاختطار النسبي: 0.66 (بنسبة أرجحية مقدارها 95‏%: 0.38‏-1.13)، الاختطار النسبي للوفيات: 1.01 (بنسبة أرجحية مقدارها 95‏%: 0.67‏-1.52) وبالنسبة لفشل العلاج كان الاختطار النسبي: 1.07 (بنسبة أرجحية مقدارها 95%: 0.48‏-2.40). أوردت اثنتان من ضمن ثلاث دراسات لتقييم تكاليف الرعاية الصحية عن انخفاض تكاليف نماذج الرعاية اللامركزية عن النماذج المركزية.

**الاستنتاج:**

كان نجاح العلاج أكثر احتمالًا بين المرضى الذين يعانون من السل المقاوم للأدوية المتعددة والذين تم علاجهم باستخدام أسلوب لامركزي. يلزم إجراء مزيد من الدراسات لاستكشاف فعالية الرعاية اللامركزية للسل المقاوم للأدوية المتعددة (MDR) في مجموعة من البيئات المختلفة. ****

## 摘要

**目的:**

对比集中治疗方法评估耐多药结核病 (MDR) 患者的分散化治疗和护理的效果。

**方法:**

我们搜索了 ClinicalTrials.gov、Cochrane 图书馆、Embase®、谷歌学术、LILACS、PubMed®、Web of Science 和世界卫生组织临床试验门户网站上有关耐多药结核病 (MDR) 患者的分散化和集中治疗结果的研究。 主要的成果是治疗成功。 条件允许时，我们还对死亡率、失访率、治疗依从性和卫生系统成本进行了评估。 为了获取汇总相对风险 (RR) 估值，我们进行了随机效应元分析。

**结果:**

八项研究符合纳入评审的资格标准。 共有 4026 名参与者参与了六项队列研究，报告了治疗结果。 治疗成功的分散化和集中护理的汇总 RR 估值为 1.13 (95% CI: 1.01–1.27)。 相应的失访率 RR 估值为： 0.66 (95% CI: 0.38–1.13)，死亡率 RR 估值为： 1.01 (95% CI: 0.67–1.52)，治疗失败 RR 估值为： 1.07 (95% CI: 0.48–2.40)。 三份评估医疗护理成本的研究中，有两项指出，分散模式的护理成本低于集中模式的成本。

**结论:**

采用分散化方法进行治疗的耐多药结核病患者的治疗成功率更高。 需要开展进一步研究，以探索耐多药结核病分散化治疗在多种不同环境下的效果。

## Резюме

**Цель:**

Провести оценку эффективности децентрализованной формы оказания медицинской помощи пациентам с туберкулезом со множественной лекарственной устойчивостью (МЛУ) по сравнению с централизованными подходами.

**Методы:**

Мы провели поиск в ClinicalTrials.gov, Кокрановской библиотеке, Embase®, Google Scholar, LILACS, PubMed®, Web of Science и на портале клинических испытаний Всемирной организации здравоохранения на предмет исследований, в которых сообщается о результатах лечения туберкулеза с МЛУ при децентрализованной и централизованной форме оказания медицинской помощи. Основным результатом была эффективность лечения. По возможности мы также проводили оценку летальности, популяции пациентов, выбывших из последующего наблюдения, следования предписанному режиму и затрат в системе здравоохранения. Чтобы получить объединенные оценки отношения рисков (ОР), мы провели метаанализы с использованием модели случайных эффектов.

**Результаты:**

Восемь исследований соответствовали критериям приемлемости для включения в обзор. В шести когортных исследованиях, в которых участвовало 4026 человек, сообщалось о результатах лечения. Объединенная оценка ОР для эффективности лечения при децентрализованной и централизованной формах лечения составила 1,13 (95%-й ДИ: 1,01–1,27). Соответствующими оценками ОР для пациентов, выбывших из последующего наблюдения, были: 0,66 (95%-й ДИ: 0,38–1,13), ОР для летальности: 1,01 (95%-й ДИ: 0,67–1,52), ОР для безуспешного лечения: 1,07 (95%-й ДИ: 0,48–2,40). В двух из трех исследований, в которых проводилась оценка затрат в системе здравоохранения, сообщалось о более низких затратах при децентрализованной форме оказания медицинской помощи по сравнению с централизованной моделью.

**Вывод:**

Эффективность лечения была более вероятной среди пациентов с МЛУ-туберкулезом, получавших медицинскую помощь с использованием децентрализованного подхода. Необходимы дальнейшие исследования для изучения эффективности децентрализованного подхода при лечении туберкулеза с МЛУ в различных условиях.****

## Introduction

*Mycobacterium tuberculosis* resistant to both isoniazid and rifampicin, so-called multidrug resistance, poses a major threat to the control of tuberculosis worldwide. In 2015, there were an estimated 480 000 new cases of multidrug-resistant (MDR) tuberculosis, an additional 100 000 cases with rifampicin resistance that also required treatment with second-line medicines, and approximately 250 000 deaths from MDR tuberculosis.^1^ An estimated 9.5% of people with MDR tuberculosis have extensively drug-resistant (XDR) tuberculosis – i.e. MDR tuberculosis that is also resistant to a second-line injectable drug and a fluoroquinolone. It has been estimated that, of all the cases of MDR tuberculosis that commenced treatment in 2013, only 52% achieved treatment success and the rest died (17%), were lost to follow-up or otherwise not evaluated (22%) or were identified as treatment failures (9%).^1^ The recommended therapy for MDR tuberculosis requires a combination of second-line drugs that are, in general, more costly, less efficacious, more toxic and must be taken for much longer than the first-line drugs used against tuberculosis.^2^

Historically, treatment for MDR tuberculosis has been provided through specialized, centralized programmes and typically involved prolonged inpatient care.^3^ This approach is based on the view that treatment adherence, the management of adverse events and infection control may be better in hospital settings than in the community.^4^^,^^5^ However, in many centralized facilities, insufficient resources preclude the prolonged inpatient care of cases of MDR tuberculosis. Reliance on centralized treatment, especially in facilities that lack effective infection control and where treatment may be delayed until inpatient beds become available, may inadvertently increase the risk of transmission of MDR *M. tuberculosis*. In addition, in comparison with decentralized interventions, centralized approaches have been associated with poorer rates of retention in care.^6^ In the treatment of drug-susceptible tuberculosis, decentralized care is well established and appears as effective as hospital-based approaches.^7^^–^^9^ Since 2011, the World Health Organization (WHO) has recommended that “patients with multidrug-resistant tuberculosis should be treated using mainly ambulatory care”.^2^ This recommendation was, however, based on the results of a small number of uncontrolled studies.^2^

We recently performed a systematic review and meta-analysis to try to determine if – compared with treatment and care provided solely by specialized centres for the treatment of MDR tuberculosis – decentralized treatment and care for MDR tuberculosis patients was more or less likely to lead to improved treatment outcomes, treatment adherence, adverse events, acquired drug resistance, lower patient costs and lower health-system costs. Our results have already contributed to forthcoming, revised WHO guidelines for the treatment of tuberculosis.

## Methods

Our systematic review was conducted in accordance with the Preferred Reporting Items for Systematic Reviews and Meta-analyses guidelines.^10^

### Study eligibility

Studies were eligible if they included both patients receiving decentralized care and patients receiving centralized care – as defined below. Studies were excluded if they lacked a comparator group or enrolled fewer than 10 participants in the intervention arm. This approach enabled a direct comparison to be performed between individuals receiving either model of care in the same setting. Included studies needed to report on at least one clinical outcome – i.e. treatment adherence, the standard WHO-defined tuberculosis treatment outcomes of cure, completion, death, failure or relapse^11^ and/or adverse reactions. Studies reporting costs, to patients and/or health systems, were also included. We included case–control studies that each included at least 10 patients, modelling studies, prospective cohorts, randomized controlled trials and retrospective cohorts. Unpublished studies were sought through consultation with experts in the field and by hand-searching the International Union of Tuberculosis and Lung Disease’s database of conference abstracts,^12^ OpenSIGLE^13^ and other grey literature.

We considered patients with MDR tuberculosis to be those with a microbiological or clinical diagnosis of MDR tuberculosis – including XDR tuberculosis – that had commenced second-line drug therapy. A clinical diagnosis included contacts – exposed to patients with MDR tuberculosis – who developed signs and symptoms of tuberculosis but were not microbiologically confirmed as cases. Decentralized care was defined as treatment and care provided in the community where the patient resided – e.g. in a community centre, a peripheral health centre or the patient’s home or workplace. A key component of the definition of decentralized care was the use of non-specialized workers – e.g. community workers, treatment supporters or volunteers.^11^ Even with care that we considered decentralized, an initial period of hospitalization during the initiation of therapy was permissible, so long as the majority of care was delivered in a decentralized fashion. To be considered centralized, care had to have been provided solely by specialist centres for the treatment of MDR tuberculosis, either in such a centre – as an inpatient and/or outpatient – or in outpatient facilities near to such a centre.

Our outcomes of interest included treatment adherence, the standard WHO-defined tuberculosis treatment outcomes of cure, completion, death and failure,^11^ adverse reactions and patient and/or health-system costs.

### Search strategy

We searched for relevant publications in ClinicalTrials.gov, the Cochrane library, Embase®, Google Scholar, LILACS, PubMed®, Web of Science and the World Health Organization’s portal of clinical trials. We developed a sensitive search strategy to detect articles on MDR tuberculosis that mentioned community-based care and/or decentralized care. The search terms used with PubMed® are shown in Box 1. Searches were limited to publications published between the start of 1995, i.e. the year in which the WHO-recommended directly observed treatment, short-course (DOTS) strategy was scaled-up, and 31 May 2016. The reference lists of all articles considered relevant were searched for reports of further eligible studies. Where the findings of a study were reported in brief in one paper and then more completely in a subsequent paper, only the latter was selected for inclusion in our review. If an abstract was the only report of a potentially eligible study that we could find, we attempted to contact the abstract’s authors so that we could obtain additional information and ask the authors to complete a data-collection form. The authors of some other, fuller reports were also contacted to provide additional data, if required. Searches were not restricted by language. If eligible studies published in a language other than English were identified, these were translated by translators with experience in the field of tuberculosis.

Box 1 **Search terms used with PubMed®**

**1. Tuberculosis, Multidrug-Resistant [MeSH]**

OR

((tuberculosis OR TB) AND (multidrug-resistan* OR multidrug resistan* OR multi-drug resistan* OR “drug resistan*” OR drug-resistan* OR multiresistan* OR “multi resistan*” OR “rifampicin resistan*” OR “extensively drug-resistan*” OR “extensively-drug resistan*” OR “extensively resistan*” OR MDR OR XDR OR TDR))

OR

mdrtb OR xdr tb OR mdrtb OR mdr-tb OR xdr-tb OR tdr-tb OR “MDR TB” OR “XDR TB” OR “TDR TB”

AND

**2. (“directly observed” OR DOT OR DOTS OR DOTS-Plus OR cb-DOTS OR treatment OR “patient support”)**

AND

(community OR outpatient OR “public participation” OR community-based OR decentralized OR non-specialized OR “periph* health centres” OR home-based OR ambulatory OR clinic OR “community health worker” OR CHW OR volunteer)

### Data selection and extraction

Two reviewers independently screened all titles, abstracts and full-text articles, to identify the studies eligible for review inclusion, before two reviewers independently extracted data from all of the eligible reports. Differences between reviewers were resolved by consensus. We extracted the proportions of MDR tuberculosis patients from each study that were considered to be treatment successes, lost to follow-up, deaths or treatment failures. Other study characteristics recorded, when available, were adverse events, details about the decentralized and/or centralized care interventions, drug regimens used, health-system and patient costs, human immunodeficiency virus (HIV) prevalence, sample size and study design. We categorized the timing of the intervention, in relation to the control arm, as either concurrent or consecutive. Study quality was assessed using the Grading of Recommendations Assessment, Development and Evaluation (GRADE) method.^14^

### Data analysis

We used forest plots, created using RevMan version 5.2 (The Nordic Cochrane Centre, Copenhagen), to summarize the data for individual trials. Outcomes were estimated, as pooled proportions, using the exact binomial method^15^ and the statistical software SAS version 9.3 (SAS Institute, Cary, United States of America). We performed random-effects meta-analyses to account for between-study variability. Whenever the relevant data for an outcome of interest were available from three or more studies, we calculated the corresponding relative risk (RR) and 95% confidence interval (CI), for decentralized versus centralized care, using RevMan version 5.2 and a generalized linear mixed model, with study as a random effect. Heterogeneity between studies was evaluated as the *I*^2^ statistic.^16^^,^^17^ We planned to assess publication bias, using a funnel plot, if sufficient studies, i.e. at least five with the same end-point, were identified.^18^ We performed additional sensitivity analyses to explore the effect of removing the data from a study in which allocation to inpatient care had been highly selective and based on disease severity.

## Results

**Fig. 1:**
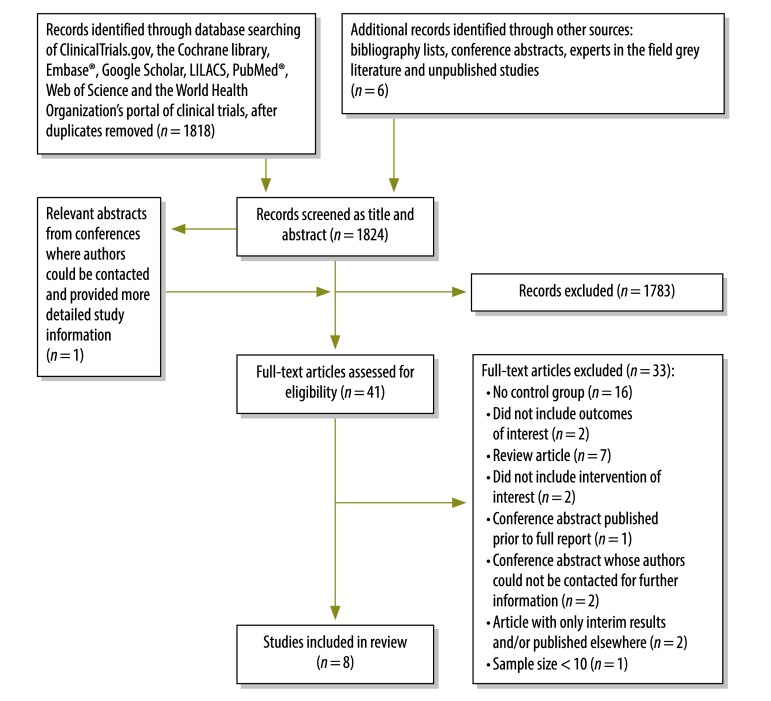
Flowchart showing the selection of studies on the centralized and decentralized care of patients with multidrug-resistant tuberculosis

Seven published studies^19^^–^^25^ and one study considered to be unpublished met the eligibility criteria for inclusion (Fig. 1; Table 1). The data for the unpublished study, which took place in Swaziland in 2016, were kindly provided by B Kerschberger (Médecins Sans Frontières, Mbabane, Swaziland), the corresponding author of a conference abstract^26^ in which the study’s initial findings were briefly summarized. Most of the excluded studies did not include a comparison group. We did not identify any relevant randomized controlled trials. Six cohort studies, with a combined total of 4026 participants, reported on treatment outcomes. Four of these were from low- or middle-income countries – i.e. the Philippines,^21^ South Africa^20^^,^^22^ and Swaziland (B Kerschberger, unpublished data), and two from middle- and high-income countries, China ^19^ and the United States of America (USA).^24^ The other two included studies were modelling studies on health-care costs.^23^^,^^25^ Of the six studies that reported on treatment outcomes, five evaluated treatment success (B Kerschberger, unpublished data),^19^^,^^20^^,^^22^^,^^24^ four evaluated loss to follow-up (B Kerschberger, unpublished data),^20^^–^^22^ four evaluated death (B Kerschberger, unpublished data),^20^^,^^22^^,^^24^ and three evaluated treatment failure(B Kerschberger, unpublished data).^20^^,^^22^ Decentralized care in some studies was based on treatment provision in patients’ homes while in other studies it was provided via community-based clinics or, in one study,^22^ via a rural hospital. Centralized care was provided in specialized hospitals, except in the unpublished study from Swaziland, in which home-based directly observed therapy provided by trained community volunteers was compared with clinic-based centralized care provided by nurses. Most decentralized and centralized care was based on the DOTS strategy (Table 1). There was no randomization of patient selection for decentralized care. Instead, allocation of sites to the intervention or control groups was based upon patient characteristics that were considered likely to make centralized care more difficult or less successful, e.g. living far from the centralized facility.^20^^,^^22^ In four of the six cohort studies, the patients were chosen for decentralized treatment based on their residential location, their socioeconomic status and their risk factors for loss to follow-up (B Kerschberger, unpublished data).^20^^,^^22^^,^^24^ In the other two cohort studies, treatment of the intervention and control groups occurred consecutively – i.e. care was initially provided by a centralized system that was subsequently replaced with a programme of decentralized care.^20^^,^^21^ None of the studies reported on acquisition of drug resistance, patient costs or treatment adherence.

**Table 1:** Key characteristics of the eight studies included in the systematic review of decentralized versus centralized care for multidrug-resistant tuberculosis, 1994–2013

Author, year, location	Study design	Year of intervention	Sample size for intervention/control	HIV prevalence in study population (%)	Description of arms		Method of selection of intervention group	Timing of intervention		Outcomes measured
					Control	Intervention		Within treatment	Relative to control	
Loveday et al.^22^ 2015, KwaZulu-Natal, South Africa	Prospective cohort	2008–2010	736/813	75	Treatment in central specialized tuberculosis hospital	Treatment in rural hospital followed by outpatient home- or clinic-based DOT, by health workers	Based on residential location	Intensive phase^a^	Concurrent	Death, loss to follow-up, treatment failure, treatment success
Chan et al.^19^ 2013, Taiwan, China	Retrospective cohort	2007–2008	290/361	0.9	Hospital and out-patient clinics	Home- based DOT, by observers and nurses	Time period	Entire duration of treatment	Consecutive	Treatment success
Kerschberger et al.^b^ 2016, Swaziland	Retrospective cohort	2008–2013	157/298	81	Clinic-based care in which patients visited nearest health facility daily	Home-based DOT, by trained community volunteers	Based on residential location and socioeconomic status	Intensive phase	Concurrent	Cost of care, death, loss to follow-up, treatment failure, treatment success
Narita et al.^24^ 2001, Florida, USA	Retrospective cohort	1994–1997	31/39	44.3	Treatment in specialized tuberculosis hospital	Outpatient DOT and/or SAT	Selected for control if: failing treatment, needed treatment of other medical condition and/or non-adherent	Entire duration of treatment	Concurrent	Death, treatment completion
Gler et al.^21^ 2012, Philippines	Retrospective cohort	2003–2006	167/416	NR	Treatment in central hospital	Community- based DOT, by trained health-care workers	Time period	After sputum-culture conversion	Consecutive	Loss to follow-up
Cox et al.^20^ 2014, Khayelitsha, South Africa	Retrospective cohort	2008–2010	512/206	72	Hospital-based care	Community-based care integrated into existing primary care tuberculosis and HIV services.	Based on residential location	Entire duration of treatment	Consecutive	Death, loss to follow-up, treatment failure, treatment success
Musa et al.^23^ 2016, Nigeria	Modelling	N/A	N/A	NR	Hospital-based care	Home-based DOT, by trained health-care providers	Random selection	Intensive phase	N/A	Health-system costs
Sinanovic et al.^25^ 2015, Khayelitsha, South Africa	Modelling	N/A	467^c^	72	Fully hospitalized model in which patients stay in hospital until culture conversion	A model of fully decentralized care in primary health-care clinics, plus other models of partially decentralized care	N/A	Entire duration of treatment	N/A	Health-system costs

DOT: directly observed therapy; HIV: human immunodeficiency virus; N/A: not applicable; NR: not reported; SAT: self-administered therapy; USA: United States of America.

^a^ Intensive phase defined by inclusion of an injectable antibiotic in the treatment regimen.

^b^ Unpublished study from Médecins Sans Frontières, Mbabane, Swaziland, 2016.

^c^ Total number of patients used in four different models of multidrug-resistant tuberculosis care.

**Fig. 2:**
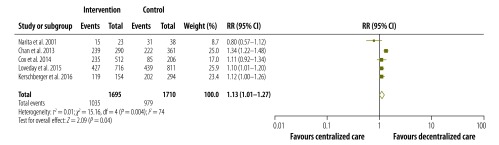
Relative risks for treatment success following the decentralized care of multidrug-resistant tuberculosis – compared with centralized care, 1994–2013

CI: confidence interval; df: degrees of freedom; RR: relative risk.

Notes: This forest plot summarizes the main results of a random-effects meta-analysis of the data from five studies. To be considered a treatment success, a patient had to show no evidence of failure after completing treatment recommended by national policy.^11^

**Fig. 3:**
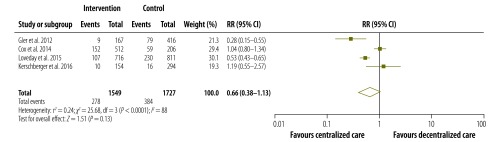
Relative risks for loss to follow-up during the decentralized care of multidrug-resistant tuberculosis – compared with centralized care, 2003–2013

CI: confidence interval; df: degrees of freedom; RR: relative risk.

Notes: This forest plot summarizes the main results of a random-effects meta-analysis of the data from four studies. A patient whose treatment was interrupted for at least two consecutive months was considered lost to follow-up.^11^

**Fig. 4:**
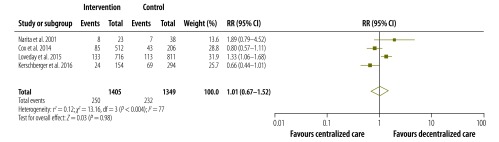
Relative risks for death during the decentralized care of multidrug-resistant tuberculosis – compared with centralized care, 1994–2013

CI: confidence interval; df: degrees of freedom; RR: relative risk.

Notes: This forest plot summarizes the main results of a random-effects meta-analysis of the data from four studies. Death was the treatment outcome recorded for any patients who died, for any reason, during the course of treatment.^11^

**Fig. 5:**
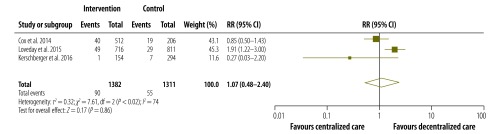
Relative risks for treatment failure following the decentralized care of multidrug-resistant tuberculosis – compared with centralized care, 2008–2013

CI: confidence interval; df: degrees of freedom; RR: relative risk.

Notes: This forest plot summarizes the main results of a random-effects meta-analysis of the data from three studies. Treatment failure was the outcome recorded when – because of a lack of conversion by the end of the intensive phase, bacteriological reversion in the continuation phase after conversion to negative, evidence of additional acquired resistance to fluoroquinolones or second-line injectable drugs or adverse drug reactions – treatment was terminated or there was a need for a permanent regimen change involving at least two anti-tuberculosis drugs.^11^

The pooled proportions of each treatment outcome are shown, separately for decentralized and centralized care, in Table 2. Overall, treatment success was achieved in 67.3% (95% CI: 53.8–78.5%) of patients who received decentralized care compared with 61.0% (95% CI: 49.0–71.7%) of those treated with centralized care. Fig. 2, Fig. 3, Fig. 4 and Fig. 5 are forest plots showing the RRs for the various treatment outcomes. Treatment success was significantly more common among those receiving decentralized care than among those in the centralized care group (RR: 1.13; 95% CI: 1.01–1.27; *I*^2^ = 74%). Although loss to follow-up was relatively less common with decentralized care than with centralized, the difference was not statistically significant (RR: 0.66; 95% CI: 0.38–1.13; *I*^2^ = 88%). The proportions of death (RR: 1.01; 95% CI: 0.67–1.52; *I*^2^ = 77%) and treatment failure (RR: 1.07; 95% CI: 0.48–2.40; *I*^2^ = 74%) with decentralized care were similar to those observed with centralized care. Owing to the small number of eligible studies, we could not assess publication bias formally.

**Table 2:** Proportions of patients with multidrug-resistant tuberculosis who achieved treatment success after receiving decentralized and centralized care, five countries, 1994–2013

Study	Centralized care						Decentralized care				
	Total patients	No. of patients (%)					Total patients	No. of patients (%)			
		Treatment success**^a^**	Loss to follow-up	Death	Treatment failure			Treatment success **^a^**	Loss to follow-up	Death	Treatment failure
Chan et al.^19^	361	222 (61.5)	ONA	ONA	ONA		290	239 (82.4)	ONA	ONA	ONA
Cox et al.^20^	206	85 (41.3)	59 (28.6)	43 (20.9)	19 (9.2)		512	235 (45.9)	152 (29.7)	85 (16.6)	40 (7.8)
Kerschberger et al.^b^	294	202 (68.7)	16 (5.4)	69 (23.5)	7 (2.4)		154	119 (77.3)	10 (6.5)	24 (15.6)	1 (0.6)
Loveday et al.^22^	811	439 (54.1)	230 (28.4)	113 (13.9)	29 (3.6)		716	427 (59.6)	107 (14.9)	133 (18.6)	49 (6.8)
Narita et al.^24^	38	31 (81.6)	ONA	7 (18.4)	ONA		23	15 (65.2)	ONA	8 (34.8)	ONA
Gler et al.^21^	416	ONA	79 (19.0)	ONA	ONA		167	ONA	9 (5.4)	ONA	ONA
**All six studies**											
Outcome/ total no. of patients (pooled percentage; 95% CI)	2126	979/1710 (61.0; 49.0–71.7)	384/1727 (18.0; 9.3–31.8)	232/1349 (18.6; 14.5–23.6)	55/1311 (4.3; 2.3–8.1)		1862	1035/1695 (67.3; 53.8–78.5)	278/1549 (11.9; 5.7–23.3)	250/1405 (17.8; 15.9–19.9)	90/1382 (4.2; 1.4–11.9)

CI: confidence interval; ONA: outcome not assessed.

^a^ Includes treatment completion and cure.^11^

^b^ Unpublished study from Médecins Sans Frontières, Mbabane, Swaziland, 2016.

In terms of the method of assigning patients to the intervention and control groups, one study differed markedly from the other included studies. In this one study,^24^ only patients who were failing treatment or non-adherent were selected for care in a specialized tuberculosis hospital. However, when, in a sensitivity analysis, we excluded data from this one study, our estimates of RRs remained largely unchanged.

Three studies, i.e. both modelling studies^23^^,^^25^ and the unpublished cohort study, reported on the health-system costs associated with decentralized and centralized care (Table 3). In both the modelling studies, from Nigeria^23^ and South Africa,^25^ decentralized care appeared to offer substantial cost savings compared with centralized care. In the retrospective cohort study from Swaziland (B Kerschberger, unpublished data), however, the estimated treatment costs with centralized care appeared very similar to those with decentralized care.

**Table 3:** Estimated health-system costs for treatment of patients with multidrug-resistant tuberculosis receiving decentralized and centralized care

Study	Study design	Country	Decentralized care			Centralized care		Difference in per-patient costs of centralized care**^a^**
			Description	Cost (US$/patient)		Description	Cost (US$/patient)	
Musa et al.^23^	Modelling of costs from a health-systems perspective	Nigeria	Home-based care for entire duration of treatment	1535		Hospital-based care for intensive phase, then home-based care for continuation phase	2095	37% higher
Sinanovic et al.^25^	Modelling of costs from a health-systems perspective	South Africa	Primary health-care clinic for entire duration of treatment	7753^b^		Hospital-based care for intensive phase – until 4-month culture conversion – then clinic-based care	13 432^c^	42% higher
Kerschberger et al.^d^	Retrospective cohort study	Swaziland	Home-based care for entire duration of treatment	13 361		Clinic-based care for intensive phase, then home-based care for continuation phase	13 006	3% lower

US$: United States dollars.

^a^ Compared with corresponding costs of decentralized care.

^b^ 95% confidence interval: 6917–8522.

^c^ 95% confidence interval: 11 165–15 494.

^d^ Unpublished study from Médecins Sans Frontières, Mbabane, Swaziland, 2016.

According to the GRADE criteria,^14^ the overall quality of the studies we used to estimate RRs was very low – mainly because the studies were observational and considerable heterogeneity existed between them (available from corresponding author).

## Discussion

In the treatment of patients with MDR tuberculosis, according to our meta-analysis, decentralized care appears to be more likely than centralized care to lead to treatment success. The loss to follow-up with decentralized care was lower – although not significantly lower – than with centralized care and the rates of death and treatment failure appeared unaffected by the type of care provided. Furthermore, from a health-system perspective, the decentralized approaches appeared either cost-neutral or cost-saving when compared with the centralized approaches.

There may be several explanations why, compared with centralized care, decentralized care was more likely to lead to treatment success. For example, although the small number of eligible studies limited the power of our meta-analysis, there is a hint that retention in care may be generally greater, or, at least, loss to follow-up may be generally rarer, when services are delivered locally. It seems likely that the delivery of care in the community could eliminate some of the barriers to treatment adherence that are encountered with often-more-distant centralized care. For example, the costs of hospitalization to patients and, often, their families may be prohibitive even if the tuberculosis treatment itself is provided free of charge.^27^ Local delivery of care may also facilitate greater support from patients’ families and their wider social networks, which may, in turn, increase the likelihood of adherence. We need further studies to examine the effect of decentralized MDR tuberculosis care on treatment adherence and patient attitudes to care.

All three studies on health-system costs that we included in our review were conducted in resource-poor low- or middle-income settings. Their conclusions – that decentralized care was cheaper or as cheap as centralized care – may not apply to high-income settings, where the costs of community-based care may be at least as high as those of centralized care. Further research is required to evaluate the impact of decentralized approaches on the costs to patients of treatment for MDR tuberculosis. The impact of such approaches on the elimination of catastrophic health expenditure for the households of such patients, which is one of the key targets of the WHO End TB Strategy,^28^ also needs to be investigated.

The strengths of our review include the comprehensive search of bibliographic databases and other information sources and our use of strict eligibility criteria to limit the review to studies in which cohorts receiving decentralized care were compared with those, from the same study population, receiving centralized care. The eligibility criteria we used, which reduced the risk of bias due to indirectness, were narrower than those used in previous systematic reviews on the care of patients with MDR tuberculosis.^29^^,^^30^

Our review also had several limitations. Substantial heterogeneity was observed between the included studies. This probably reflects the diversity in the study settings, patient populations and interventions involved. However, the effect estimates from every study in a tuberculosis-endemic setting that we included in our meta-analysis indicated that decentralized care was better – at least in terms of the probability of treatment success – than centralized care. The only study included from a low-prevalence country – i.e. the United States – indicated the opposite: that the probability of treatment success was lower with decentralized care than with centralized. Given the absence of randomized controlled trials, the frequent use of historical controls and the large level of heterogeneity between the studies, it is perhaps not surprising that we found that the overall quality of the studies we used to estimate RRs was categorized as low. This low quality places some limitations on the precision and generalizability of the results of our meta-analysis and underpins the importance of further research into the benefits of decentralized care for MDR tuberculosis in different settings. In countries where tuberculosis is endemic and national programmes increasingly adopt decentralized approaches for managing patients with MDR tuberculosis, the programmes’ interventions and outcomes need to be carefully and thoroughly reported. For future research in this field, before-and-after studies or pragmatic randomized studies – e.g. stepped-wedge cluster randomized studies – may be good choices. Well-designed operational research may enable programmes to evaluate the effectiveness of alternative approaches, in their local settings, accurately.
